# Supplementary material for: Nationwide Screening for Arthropod, Fungal, and Bacterial Pests and Pathogens of Honey Bees: Utilizing Environmental DNA from Honey Samples in Australia
Source: Insects. 2025 Jul 25;16(8):764. doi: 10.3390/insects16080764 (PMC12386956; doi:10.3390/insects16080764)
Supplement: Supplementary file 1 [file insects-16-00764-s001.zip › insects-3679320-supplementary.pdf]

Supplementary Figure S1. Map of Australia with the total number of hives in each state and the number of sample collected from each state.

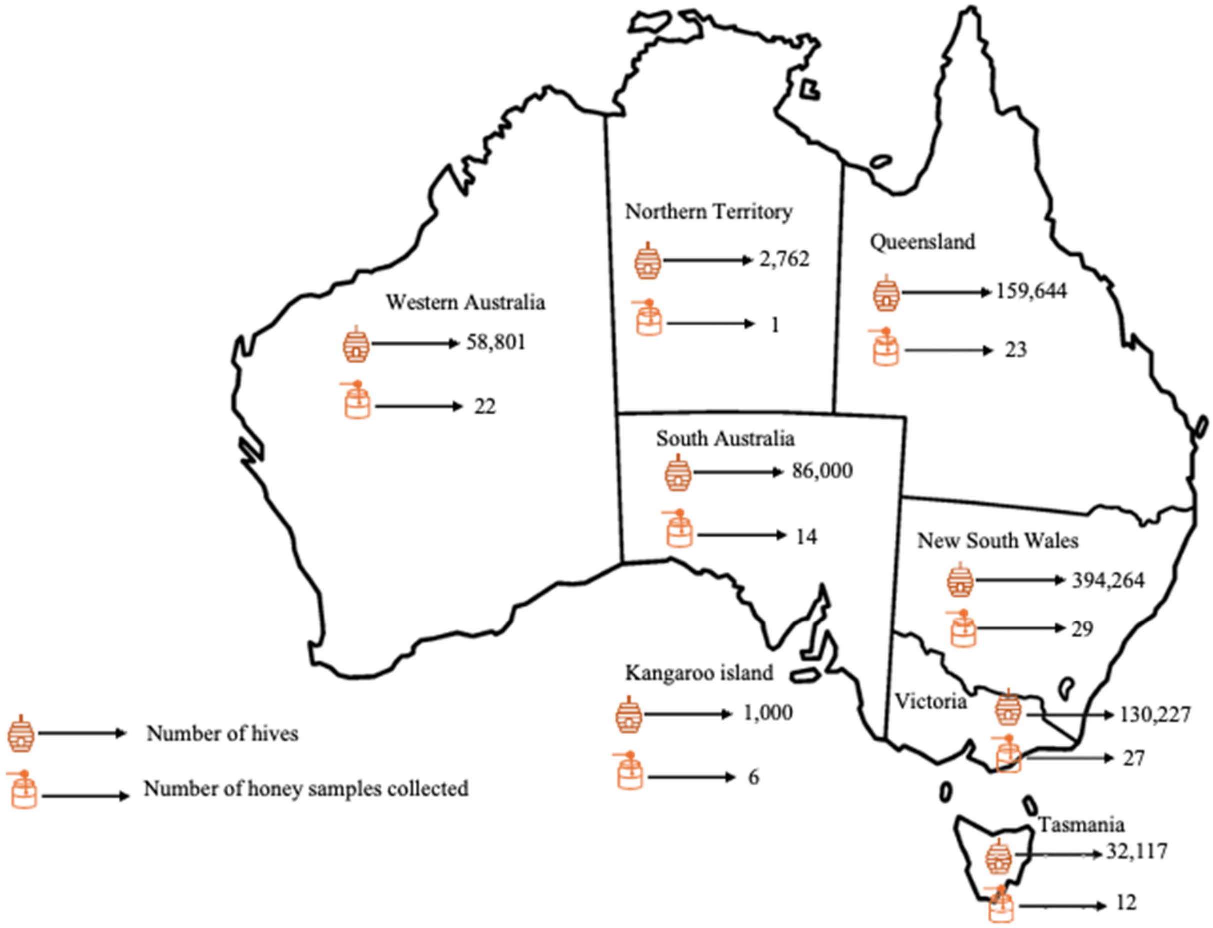

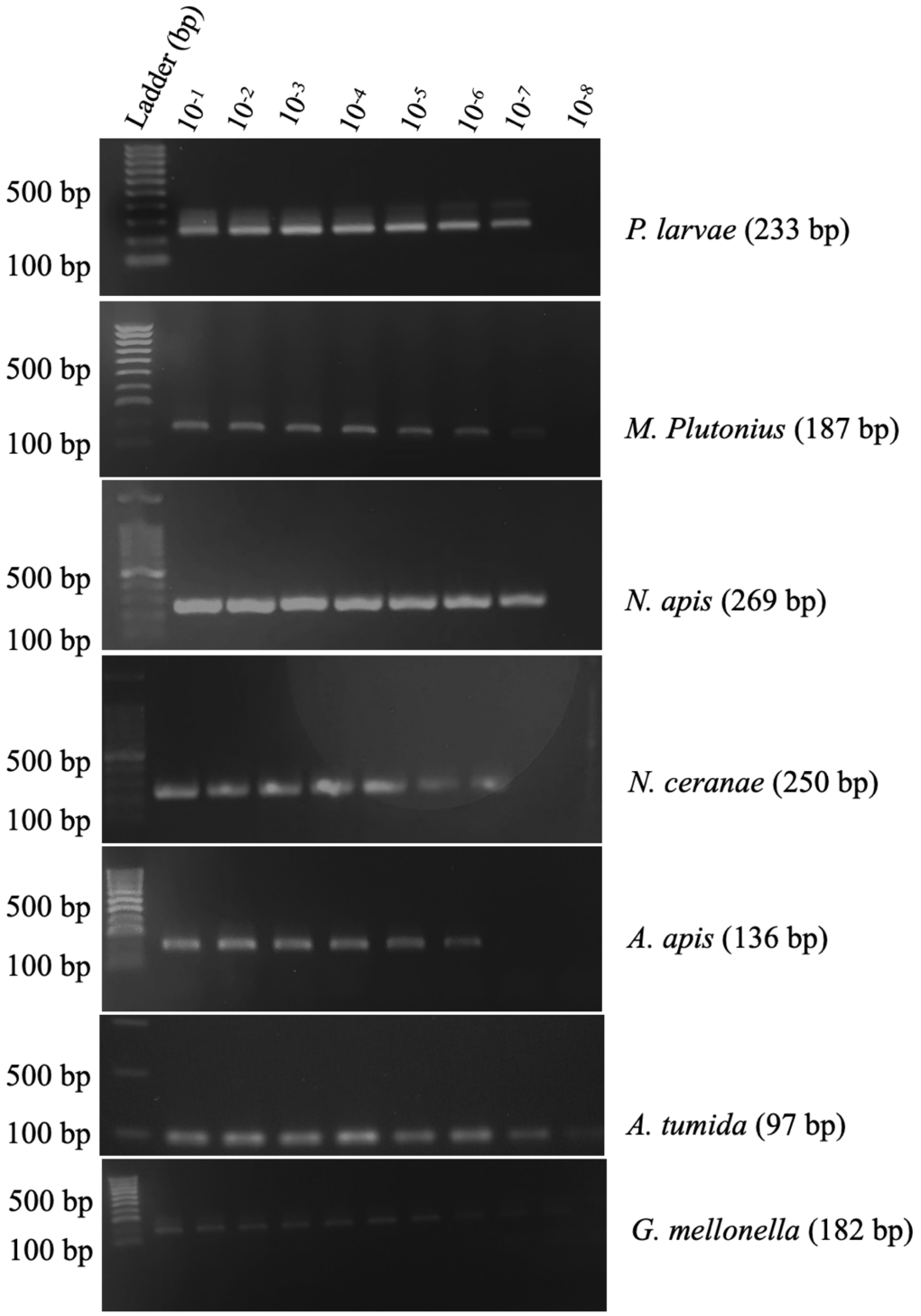

Supplementary Figure S2. Sensitivity of the chosen primer pair for the target pathogen. Each image of the agarose gel illustrates the assay's capability to amplify the target species across different DNA concentrations from 10^{-1} to 10^{-8} ng/µL.

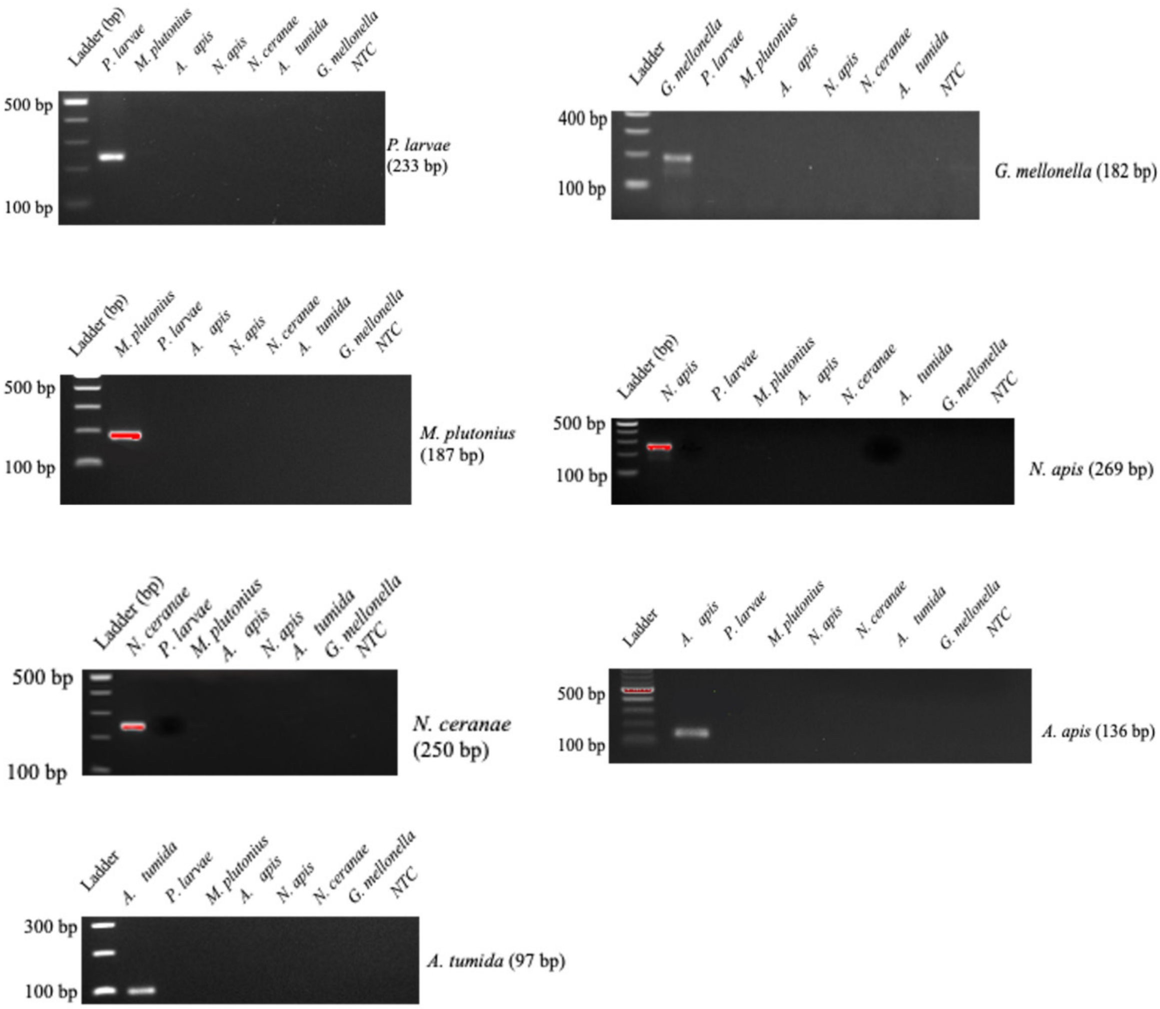

Supplementary Figure S3. The gel images show amplicons generated by pathogen-specific primers using various DNA templates. NTC refers to the no template control.

Supplementary Table S1. Honey sample metadata including sample ID, collection state, and detection status for each honey bee pathogen and pest. Presence or absence of each organism is indicated by "Yes" (positive) or "No" (negative) for each sample.

Sample Number	State	*Paenibacillus larvae*	*Melissococcus plutonius*	*Ascosphaera apis*	*Nosema apis*	*Nosema ceranae*	*Aethina tumida*	*Galleria mellonella*
1	South Australia	No	Yes	No	Yes	Yes	Yes	Yes
2	Victoria	No	No	No	No	Yes	Yes	No
3	Victoria	Yes	Yes	No	Yes	Yes	No	No
4	Victoria	No	No	No	No	No	No	No
5	South Australia	No	No	No	No	Yes	No	No
6	Victoria	Yes	Yes	No	No	No	No	No
7	Victoria	No	No	No	No	No	Yes	No
8	Queensland	Yes	No	No	No	No	No	No
9	Victoria	No	No	No	Yes	Yes	Yes	No
10	Victoria	No	No	No	No	Yes	Yes	Yes
11	Victoria	No	No	No	No	No	No	No
12	Victoria	No	No	No	No	Yes	Yes	Yes
13	Tasmania	No	No	No	Yes	Yes	No	Yes
14	Tasmania	No	No	No	No	No	No	Yes
15	Tasmania	Yes	No	No	Yes	Yes	Yes	Yes
16	Tasmania	No	Yes	Yes	Yes	No	Yes	Yes
17	West Australia	No	No	No	No	No	No	Yes
18	West Australia	No	No	No	No	No	No	Yes
19	Tasmania	No	Yes	Yes	Yes	Yes	Yes	Yes
20	Tasmania	No	No	No	No	Yes	No	Yes
21	Northern Territory	No	No	No	No	Yes	No	No
22	Tasmania	Yes	No	No	Yes	No	No	Yes
23	Tasmania	Yes	No	No	No	No	No	Yes
24	Tasmania	No	Yes	No	No	No	No	No
25	Tasmania	No	No	Yes	Yes	Yes	No	Yes

26	Tasmania	No	No	Yes	No	No	Yes	Yes
27	Kangaroo Island	No	No	No	Yes	Yes	No	No
28	Kangaroo Island	No	No	No	No	No	No	No
29	Kangaroo Island	No	No	No	Yes	No	No	No
20	Kangaroo Island	No	No	No	Yes	No	No	No
31	Victoria	Yes	Yes	No	No	Yes	Yes	Yes
32	Victoria	Yes	Yes	No	No	Yes	Yes	Yes
33	Victoria	Yes	Yes	No	No	No	Yes	Yes
34	Victoria	Yes	Yes	No	No	Yes	Yes	Yes
35	Victoria	No	No	No	No	Yes	Yes	Yes
36	New South Wales	No	No	No	No	No	No	No
37	New South Wales	No	No	No	No	No	Yes	No
38	Kangaroo Island	No	No	No	No	No	No	No
39	Kangaroo Island	No	No	No	No	No	No	No
40	Victoria	No	No	No	No	Yes	Yes	No
41	New South Wales	No	No	No	No	No	No	No
42	New South Wales	Yes	Yes	No	Yes	Yes	Yes	Yes
43	New South Wales	Yes	Yes	Yes	No	Yes	No	No
44	New South Wales	No	No	No	No	No	No	No
45	New South Wales	Yes	Yes	Yes	No	No	Yes	Yes
46	New South Wales	No	No	No	No	Yes	Yes	No
47	Queensland	Yes	No	No	No	Yes	Yes	No
48	New South Wales	Yes	No	No	No	Yes	Yes	No
49	South Australia	No	Yes	No	No	Yes	Yes	Yes
50	Queensland	No	No	No	No	No	Yes	No
51	Queensland	No	No	No	No	Yes	Yes	No
52	Tasmania	No	No	No	Yes	Yes	No	No
53	West Australia	No	No	No	Yes	Yes	No	Yes
54	West Australia	Yes	No	No	Yes	Yes	No	Yes

55	West Australia	No	No	No	No	No	No	No
56	West Australia	Yes	No	No	No	No	No	No
57	West Australia	No	No	No	No	Yes	No	Yes
58	West Australia	No	No	No	No	No	No	No
59	West Australia	No	No	No	No	No	No	No
60	West Australia	No	No	No	Yes	Yes	Yes	Yes
61	West Australia	No	No	No	No	No	No	Yes
62	West Australia	No	No	No	No	Yes	No	No
63	West Australia	No	No	No	No	No	No	No
64	West Australia	No	No	No	No	No	No	Yes
65	West Australia	No	No	No	No	Yes	No	Yes
66	West Australia	No	No	No	Yes	Yes	No	No
67	West Australia	No	No	No	Yes	Yes	No	No
68	West Australia	No	No	No	No	No	No	Yes
69	New South Wales	No	No	No	No	Yes	Yes	No
70	New South Wales	No	No	No	No	Yes	Yes	No
71	West Australia	No	No	No	No	Yes	No	No
72	West Australia	No	No	No	No	No	No	No
73	South Australia	No	No	No	No	Yes	Yes	No
74	South Australia	No	No	No	No	Yes	Yes	No
75	South Australia	No	No	No	No	No	No	No
76	South Australia	No	No	No	No	No	No	Yes
77	South Australia	No	No	No	Yes	Yes	Yes	Yes
78	Victoria	No	No	No	No	No	No	No
79	Queensland	Yes	No	No	No	Yes	Yes	No
80	South Australia	No	No	No	No	Yes	No	Yes
81	South Australia	No	No	No	No	No	No	No
82	South Australia	No	No	No	No	No	No	No
83	West Australia	No	No	No	No	No	No	No

84	West Australia	Yes	No	No	No	Yes	No	No
85	Victoria	No	No	No	No	Yes	Yes	No
86	Victoria	No	No	No	No	Yes	Yes	Yes
87	South Australia	No	Yes	No	No	Yes	Yes	Yes
88	South Australia	No	Yes	No	No	Yes	Yes	Yes
89	New South Wales	No	No	No	No	No	Yes	No
90	Victoria	No	No	No	No	Yes	No	No
91	Queensland	No	No	No	No	Yes	No	No
92	New South Wales	No	No	No	No	Yes	No	No
93	New South Wales	No	No	No	No	Yes	No	No
94	New South Wales	No	No	No	Yes	Yes	No	No
95	New South Wales	Yes	No	No	No	No	No	No
96	New South Wales	No	No	No	No	Yes	No	No
97	New South Wales	No	No	No	No	Yes	No	No
98	New South Wales	No	No	No	No	Yes	No	No
99	New South Wales	No	No	No	No	Yes	No	Yes
100	New South Wales	No	No	No	No	Yes	No	No
101	New South Wales	Yes	Yes	No	Yes	Yes	No	No
102	New South Wales	No	No	No	No	No	No	No
103	New South Wales	No	No	No	No	No	No	No
104	New South Wales	No	No	No	No	No	No	No
105	New South Wales	No	No	No	No	Yes	No	No
106	New South Wales	No	No	No	No	Yes	No	No
107	Victoria	Yes	Yes	Yes	Yes	Yes	No	Yes
108	Victoria	Yes	Yes	No	Yes	Yes	No	Yes
109	Victoria	No	No	No	No	Yes	No	No
110	Victoria	No	No	No	No	Yes	No	No
111	South Australia	No	No	No	No	No	Yes	Yes
112	New South Wales	Yes	No	No	No	No	Yes	No

113	New South Wales	Yes	No	No	No	No	No	No
114	Victoria	No	No	No	No	No	No	No
115	Victoria	No	Yes	No	No	Yes	Yes	Yes
116	Victoria	Yes	Yes	No	Yes	Yes	Yes	Yes
117	Victoria	No	No	No	No	No	No	No
118	Queensland	Yes	Yes	No	No	No	Yes	No
119	Queensland	Yes	No	No	No	No	Yes	No
120	Queensland	No	Yes	No	No	Yes	Yes	Yes
121	Queensland	No	No	No	No	Yes	Yes	No
122	Queensland	No	No	No	No	No	Yes	No
123	Queensland	No	Yes	No	No	Yes	Yes	No
124	Queensland	Yes	No	No	No	No	No	No
125	Queensland	No	No	No	No	Yes	Yes	Yes
126	Queensland	No	No	No	No	No	Yes	No
127	Queensland	No	No	No	No	Yes	Yes	Yes
128	Queensland	No	No	No	No	Yes	Yes	Yes
129	Queensland	No	No	No	No	Yes	Yes	Yes
130	Queensland	No	No	No	No	No	Yes	No
131	Queensland	No	No	No	No	Yes	Yes	Yes
132	Queensland	No	No	No	No	Yes	No	No
133	Queensland	No	No	No	No	No	No	No
134	Queensland	No	No	No	No	No	No	No
135	Queensland	No	No	No	No	Yes	No	No
		**28**	**24**	**7**	**25**	**77**	**54**	**49**

Supplementary Table S2: Probability table and effect sizes for honey bee pathogens/pests co-occurrence, based on a probabilistic species co-occurrence matrix. The table includes the following parameters: **pathogen/pest 1_inc** (Incidence/number of samples positive for pathogen/pest 1), **pathogen/ pest 2_inc** (Incidence/number of samples positive for pathogen/pest 2), **obs_cooccur** (observed number of samples where both pathogens/pests are present), **prob_cooccur** (probability of both pathogens/pests occurring in a sample), **exp_cooccur** (expected number of samples with both pathogens/pests under independent distribution), **p_lt** (probability that the observed co-occurrence is lower than expected under an independent distribution), **p_gt** (probability that the observed co-occurrence is higher than expected under an independent distribution), and **effect_size** (the magnitude of deviation between observed and expected co-occurrence, indicating the strength of association between the two pathogens/pests)

Pathogen/Pest 1	Pathogen/Pest 2	Incidence of Pathogen/Pest 1_inc	Incidence of Pathogen/Pest 2_inc	Obs_cooccur	Prob_cooccur	exp_cooccur	p_lt	p_gt	effects size
P. larvae	*M. plutonius*	28	24	14	0.037	5.0	1.00	0.00001	0.0666
P. larvae	*A. apis*	28	7	3	0.011	1.5	0.96	0.15581	0.0111
P. larvae	*N. apis*	28	25	9	0.041	5.6	0.97	0.06581	0.0251
P. larvae	*N. ceranae*	28	77	15	0.115	15.6	0.48	0.67507	-0.0044
P. larvae	*A. tumida*	28	54	13	0.083	11.2	0.84	0.28489	0.0133
P. larvae	*G. mellonella*	28	49	13	0.075	10.2	0.92	0.15120	0.0207
M. plutonius	*A. apis*	24	7	5	0.009	1.2	0.99	0.00198	0.0281
M. plutonius	*N. apis*	24	25	9	0.036	4.8	0.99	0.02280	0.0311
M. plutonius	*N. ceranae*	24	77	18	0.099	13.3	0.99	0.02776	0.0348
M. plutonius	*A. tumida*	24	54	17	0.071	9.6	0.99	0.00082	0.0548
M. plutonius	*G. mellonella*	24	49	17	0.065	8.7	0.99	0.00017	0.0614
A. apis	*N. apis*	7	25	4	0.010	1.4	0.99	0.02949	0.0192
A. apis	*N. ceranae*	7	77	4	0.029	3.9	0.67	0.62275	0.0007
A. apis	*A. tumida*	7	54	4	0.021	2.8	0.90	0.28532	0.0088
A. apis	*G. mellonella*	7	49	6	0.019	2.5	0.99	0.00931	0.0259
N. apis	*N. ceranae*	25	77	21	0.111	15.0	0.99	0.00752	0.0444
N. apis	*A. tumida*	25	54	9	0.080	10.8	0.28	0.84380	-0.0133
N. apis	*G. mellonella*	25	49	15	0.073	9.8	0.99	0.01889	0.0385
N. ceranae	*A. tumida*	77	54	39	0.222	30.0	0.99	0.00120	0.0666
N. ceranae	*G. mellonella*	77	49	35	0.202	27.2	0.99	0.00407	0.0577
A. tumida	*G. mellonella*	54	49	29	0.145	19.6	0.99	0.00058	0.0696
